# Supplementary material for: Atomic resolution structure of serine protease proteinase K at ambient temperature
Source: Sci Rep. 2017 Mar 31;7:45604. doi: 10.1038/srep45604 (PMC5374539; doi:10.1038/srep45604)
Supplement: Supplementary Information [file srep45604-s1.pdf]

Supplementary Information

Atomic resolution structure of serine protease proteinase K at ambient temperature

Tetsuya Masuda, Mamoru Suzuki, Shigeyuki Inoue, Changyong Song, Takanori Nakane, Eriko Nango, Rie Tanaka, Kensuke Tono, Yasumasa Joti, Takashi Kameshima, Takaki Hatsui, Makina Yabashi, Bunzo Mikami, Osamu Nureki, Keiji Numata, So Iwata & Michihiro Sugahara

a

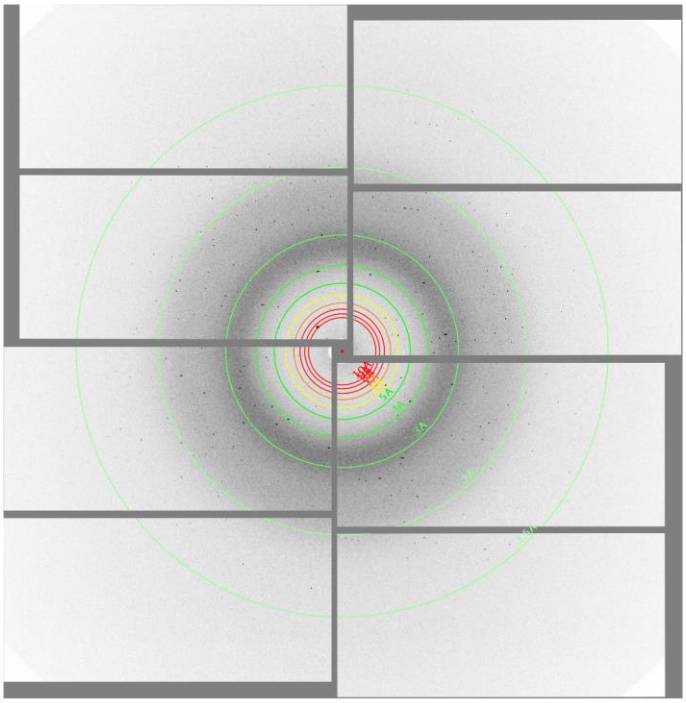

b

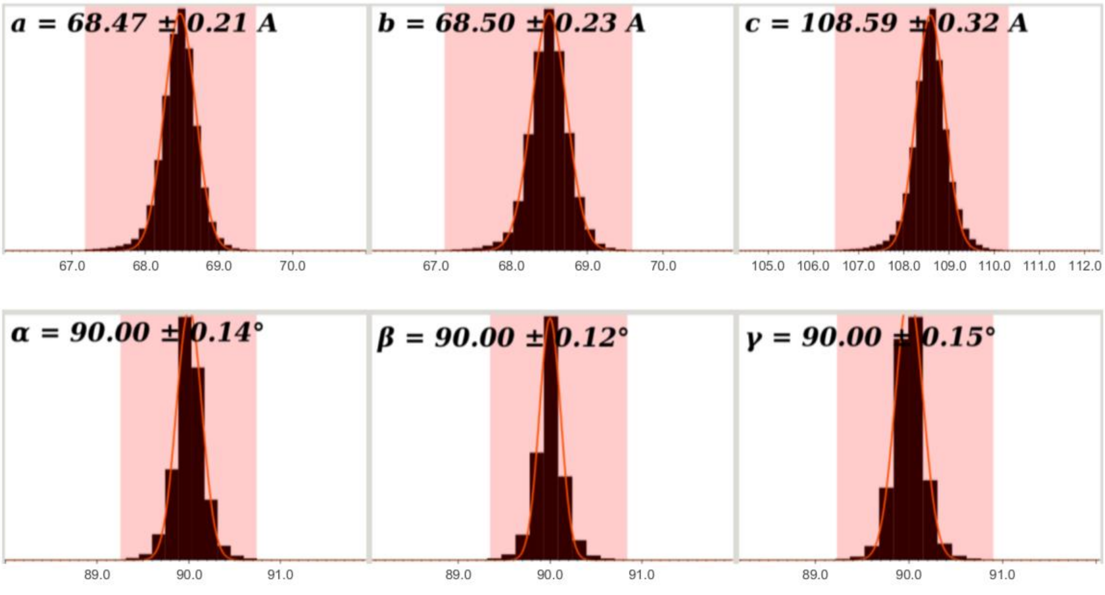

Supplementary Figure 1 | A diffraction pattern and the distributions of unit cell parameters. (**a**) A typical XFEL single diffraction pattern from an individual proteinase K microcrystal in hydroxyethyl cellulose. (**b**) The distributions of unit cell parameters resulting from processing a series of diffraction patterns.

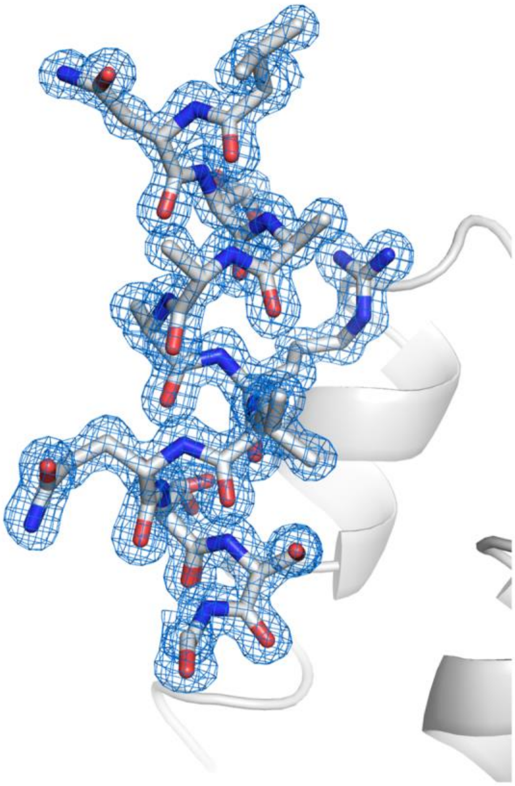

Supplementary Figure 2 | A close-up view of the proteinase K structure with a 2mF_o–DF_c electron-density map contoured at the 1.5σ level.

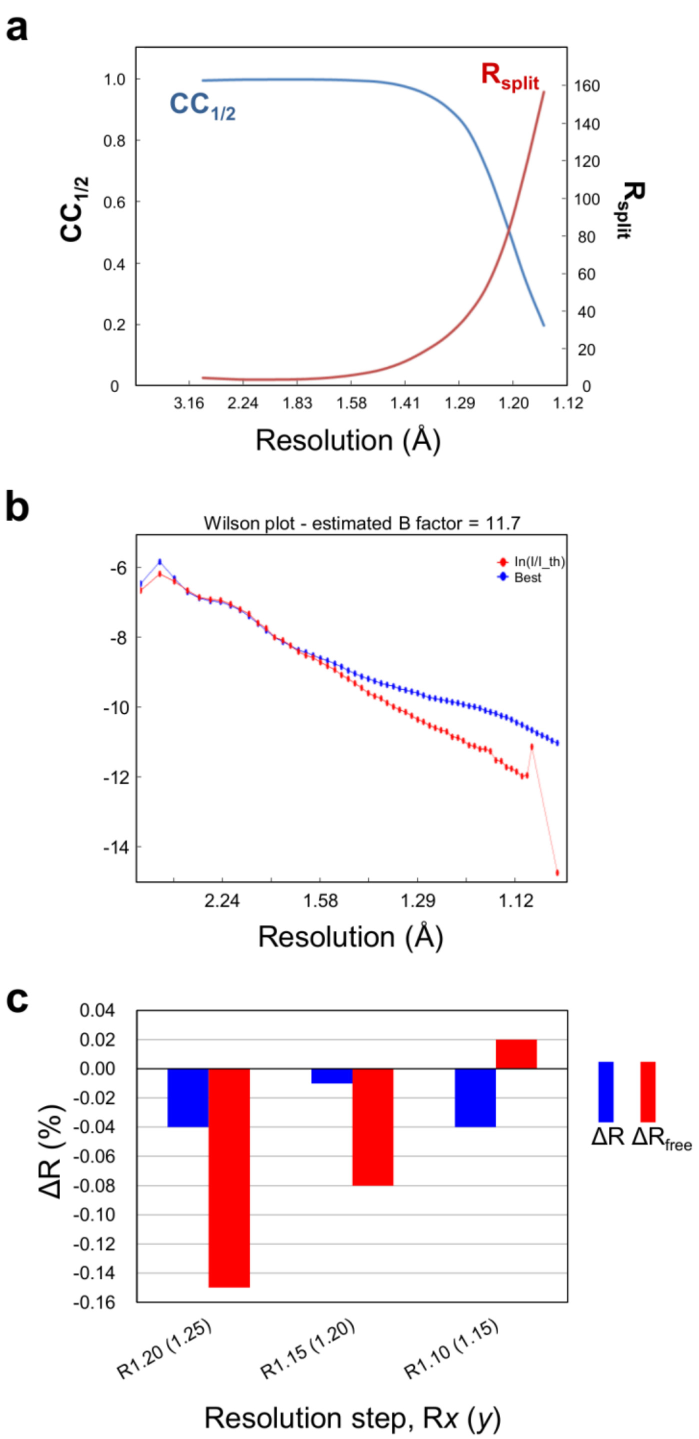

Supplementary Figure 3 | 'High resolution limit' of SFX structure. (**a**) Plots of $CC_{1/2}$ and R_{split} against resolution. (**b**) Wilson plot. (**c**) Results of paired refinement. R*x* (*y*) means that *R* values calculated at *y* Å resolution with the model refined at *y* Å resolution were compared with those using the model refined at *x* Å resolution.

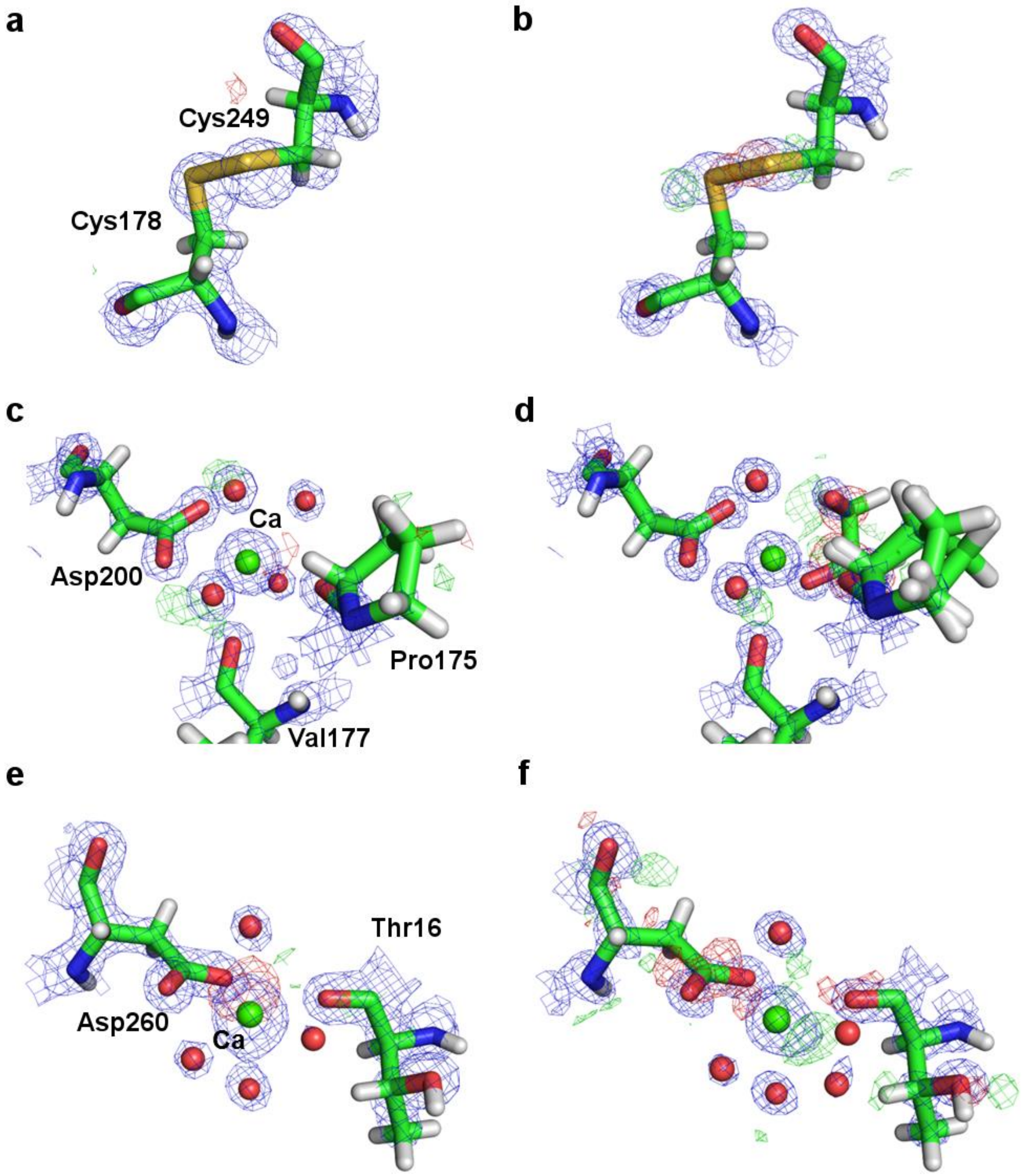

Supplementary Figure 4 | Comparison of SFX and SRX in disulfide linkage as well as Ca binding sites that are susceptible to radiation damage. Difference maps of disulfide linkages between Cys178 and Cys249 in (**a**) SFX structure and (**b**) SRX structure. No negative and/or positive peaks are detected in the SFX structure, whereas both negative and positive peaks are observed in the SRX structure. Electron density maps around two Ca-binding sites in SFX (**c,e**) and SRX (**d,f**). Unusual peaks were not detected in the SFX structure, whereas, some unusual negative peaks were found in the SRX structure. The σA-weighted 2mF_o–DF_c maps contoured at 2.0σ (**a,b,e,f**) or 3.0σ (**c,d**) are shown in blue and mF_o–DF_c maps contoured at 3.0σ and -3.0σ are shown in green and red, respectively.

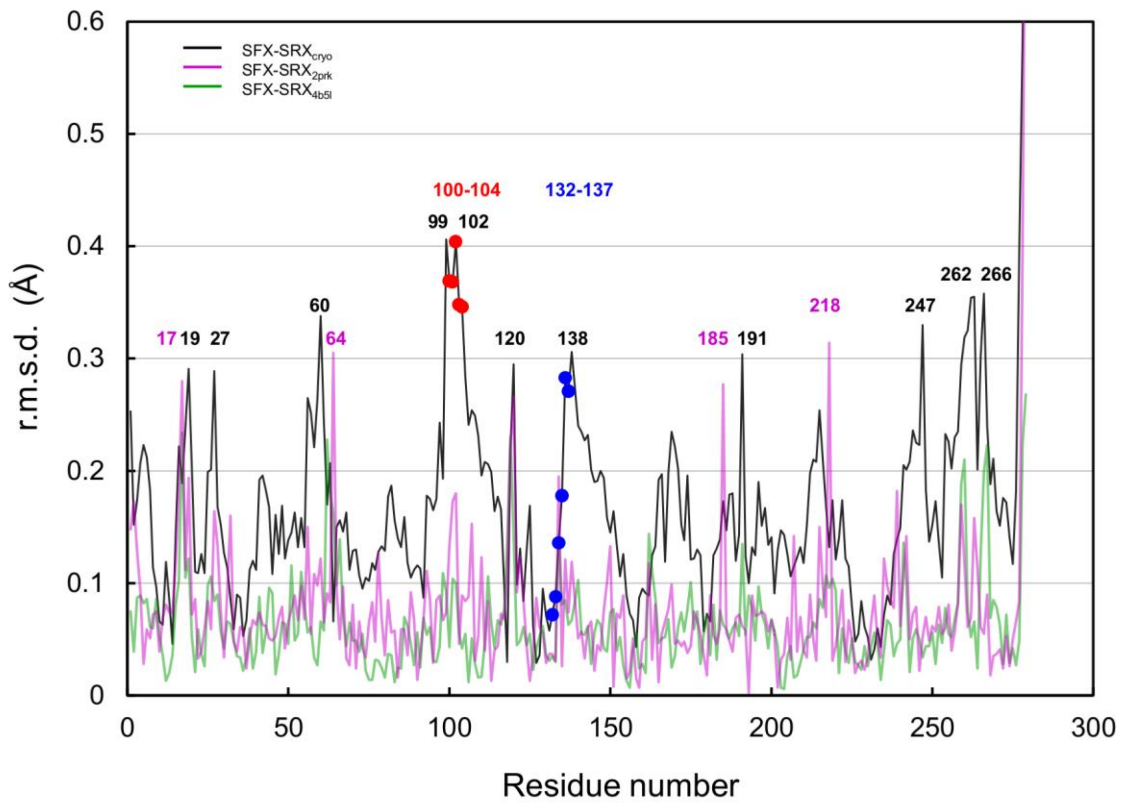

Supplementary Figure 5 | R.m.s.d. differences between SFX and SRX structures. The differences between SFX and SRX at cryo temperature (black line), (**b**) between SFX and SRX (2prk) at room temperature (pink line), and between SFX and SRX (4b5l) at room temperature (green line). Structures of SFX and SRX are superposed and the main-chain r.m.s.d, value was plotted against the residue number. One of the substrate binding sites (Gly100–Tyr104) is shown in red, and the other substrate binding site, Ser132–Tyr137, is shown in blue.

Supplementary Table 1 |Statistics for the processing of the diffraction data for SFX.

Center 1/nm	# refs	Possible	Compl	Meas	Red	SNR	Std dev	Mean	d(A)	Min 1/nm	Max 1/nm
1.874	5826	5826	100	16202738	2781.1	23.16	4524.90	4840.32	5.34	0.368	3.380
3.819	5536	5536	100	10183529	1839.5	27.79	2341.81	2154.56	2.62	3.380	4.258
4.566	5487	5487	100	8217270	1497.6	28.77	1431.20	1325.65	2.19	4.258	4.874
5.119	5418	5418	100	6913463	1276.0	26.25	764.77	715.85	1.95	4.874	5.364
5.571	5412	5412	100	6716219	1241.0	22.66	388.49	362.21	1.79	5.364	5.778
5.959	5401	5401	100	6373132	1180.0	18.20	229.76	211.9	1.68	5.778	6.140
6.302	5371	5371	100	6056897	1127.7	14.52	150.22	143.39	1.59	6.140	6.464
6.611	5377	5377	100	5824101	1083.2	11.06	100.54	95.25	1.51	6.464	6.758
6.893	5353	5353	100	5602944	1046.7	7.90	65.27	61.45	1.45	6.758	7.029
7.154	5361	5361	100	5434722	1013.8	5.70	44.02	41.09	1.40	7.029	7.280
7.397	5344	5344	100	5252900	983.0	4.37	32.10	29.87	1.35	7.280	7.515
7.625	5312	5312	100	5001867	941.6	3.30	23.63	21.51	1.31	7.515	7.736
7.841	5357	5357	100	4236507	790.8	2.46	18.48	16.15	1.28	7.736	7.945
8.045	5334	5334	100	3043739	570.6	1.80	14.55	12.58	1.24	7.945	8.144
8.239	5307	5307	100	2243599	422.8	1.26	12.45	9.57	1.21	8.144	8.333

Supplementary Table 2 | The observed hydrogen atoms in α-helices α3–α5 in SFX and SRX structures. Hydrogen atom positions found only in SFX (colored red) and SRX (colored blue).

Hydrogen Bonds in Helix α3					
				SFX	SRX
D117	N	O	F113	H	H
S116	N	O	F113	H	-
S116	N	O	D112	H	-
A115	N	O	M111	-	-
V114	N	O	G110	-	H
F113	N	O	A109	H	H
D112	N	O	I108	-	-
M111	N	O	I107	H	H
G110	N	O	T106	-	-
A109	N	O	S105	H	H
I108	N	O	Y104	-	-
I107	N	O	Q103	-	-
T106	N	O	Q103	-	-

Hydrogen Bonds in Helix α4					
				SFX	SRX
S151	N	O	R147	H	H
S150	N	O	A146	H	-
Q149	N	O	A145	H	-
L148	N	O	A144	H	-
R147	N	O	S143	-	-
A146	N	O	N142	-	-
A145	N	O	V141	H	H
A144	N	O	S140	-	H
S143	N	O	S139	-	H
N142	N	O	S138	H	-

Hydrogen Bonds in Helix α5					
				SFX	SRX
K242	N	O	L237	H	-
G241	N	O	M238	H	H
G241	N	O	L237	H	H
L240	N	O	Y236	H	-
T239	N	O	A235	H	H
M238	N	O	A234	H	H
L237	N	O	L233	H	-
Y236	N	O	G232	H	H
A235	N	O	A231	-	H
A234	N	O	V230	-	-
L233	N	O	H229	H	H
G232	N	O	P228	-	-
